# A population-based study on prevalence and risk factors of gastroesophageal reflux disease in the Tibet Autonomous Region, China

**DOI:** 10.7717/peerj.6491

**Published:** 2019-02-28

**Authors:** Haoxiang Zhang, Wenwen Gao, Lei Wang, Suzhen, Yanming Gao, Baoli Liu, Hao Zhou, Dianchun Fang

**Affiliations:** 1 Department of Gastroenterology, Southwest Hospital, Army Medical University, Chongqing, China; 2 Department of Gastroenterology, The General Hospital of Tibet Military Region, Lhasa, Tibet, China; 3 Department of Gastroenterology, The People’s Hospital of Shigatse, Shigatse, Tibet, China; 4 Department of Clinical Laboratory, Bashan Hospital, Chongqing, China; 5 Department of Urology, The People’s Hospital of Fujian Province, Fuzhou, Fujian, China

**Keywords:** Gastroesophageal reflux disease, High altitude, Tibetan dietary habits

## Abstract

**Objective:**

To investigate the prevalence and risk factors of gastroesophageal reflux disease (GERD) in the Tibet Autonomous Region, China.

**Methods:**

In this cross-sectional study, a stratified random sampling method was used for collecting samples in the Tibet Autonomous Region. A total of 10,000 individuals were selected from October 2016 to June 2017. A previously-published, validated questionnaire including six items related to the symptoms of GERD was used for evaluating GERD. In addition, basic demographic data, lifestyle, dietary habits, medical history and family history of GERD were investigated to identify risk factors of GERD.

**Results:**

A total of 5,680 completed questionnaires were collected and analyzed. The prevalence of GERD in this area was 10.8%. Age (30–40 years vs. under 18 years, odds ratio (OR): 3.025; 40–50 years vs. under 18 years, OR: 4.484), education level (high school vs. primary, OR: 0.698; university vs. primary, OR: 2.804), ethnic group (Han vs. Tibetan, OR: 0.230; others vs. Tibetan, OR: 0.304), altitude of residence (4.0–4.5 km vs. 2.5–3.0 km, OR: 2.469), length of residence (<5 years vs. ≥5 years, OR: 2.218), Tibetan sweet tea (yes vs. no, OR: 2.158), Tibetan barley wine (yes vs. no, OR: 1.271), Tibetan dried meat (yes vs. no, OR: 1.278) and staying up late (yes vs. no, OR: 1.223) were significantly (all *P* < 0.05) and independently associated with GERD.

**Conclusions:**

The prevalence of GERD is high in the Tibet Autonomous Region, China. Geographic conditions, ethnic group and lifestyle are risk factors for GERD.

## Introduction

Gastroesophageal reflux disease (GERD), which results from refluxed gastric contents into esophagus, is a digestive disorder and leads to troublesome symptoms and/or complications such as heartburn and regurgitation (Vakil et al., 2006). GERD is common in western countries; the prevalence ranges from 11.6% to 27.8% (El-Serag et al., 2014). In the Middle East and the Indian studies, the GERD prevalence estimates are 8.7–33.1% (El-Serag et al., 2014) and 16.2–22.2% (Kumar et al., 2011; Sharma et al., 2011; Wang et al., 2016), respectively. On the other hand, although the GERD prevalence in Asia is increasing (Ho, 2008; Rajendra, Kutty & Karim, 2004), it is much lower (2.5–7.8%) in East Asia (El-Serag et al., 2014) than the Western countries, suggesting racial and geographic factors are important to GERD pathogenesis (Sharma et al., 2008).

The average altitude of the Tibet Autonomous Region in China is over 4,000 m. The average annual temperature is 0–10 °C; however, the temperature varies significantly (about 20 °C) from day to night. Tibetan barley, but not rice or wheat that is widely grown in China, is the major cereal cultivated in this region because of its hardiness, and meat dishes are principally to be yak, goat and mutton rather than pork, chicken or beef. The unique Tibetan culture and lifestyle are therefore developed based on these conditions. Although several large-scale epidemiologic studies conducted in China have been reported (Chen et al., 2005; He et al., 2010; Hu et al., 2002; Pan et al., 2008; Wong et al., 2003), the data gathered from these studies may not apply to people living under this critical geographic condition. Here, we investigated the prevalence and risk factors of GERD by questionnaire in the Tibet Autonomous Region, China. The results would be informative for prevention and treatment of GERD in this specific population.

## Materials and Methods

### Study population, questionnaire and data collection

In this cross-sectional study, we used a stratified random sampling method for collecting samples in Lhasa City, Lhoka City, Xigazê City and Cona County of the Tibet Autonomous Region, China from October 2016 to June 2017. Finally, the altitude of residence was randomly selected as a Primary Sampling Units from each stratum. A total of 10,000 individuals from 2,000,000 populations in the Tibet Autonomous Region were selected to answer a questionnaire at local hospitals/clinics.

The institutional review board of Southwest Hospital, Army Medical University has reviewed and approved this study and decided the informed consent could be waived due to the observational nature of this study.

The self-assessment questionnaire was written in Chinese and designed based on the one published by Jones et al. (2009). Briefly, six items including heartburn, regurgitation, nausea, epigastric pain, sleep disturbance because of heartburn and/or regurgitation, and medication for heartburn and/or regurgitation, were used for evaluation of GERD. Scores (zero to three points) were given according to the frequency (0, 1, 2–3 or 4–7 days) of each item over the previous week. The cut-off was set as eight points for the highest specificity and sensitivity as previously reported (Jones et al., 2009). We therefore randomly selected 80 respondents for verifying the questionnaire used in this study. The diagnostic criteria were endoscopy examination in addition to clinical symptoms (Iwakiri et al., 2016). The exclusion criteria were (1) gastrointestinal related organic lesions; (2) surgical history; (3) taking acid-suppressing drugs or non-steroidal anti-inflammatory drugs within 2 weeks; (4) secondary esophagitis caused by a variety of clear causes; (5) pregnant or lactating women; (6) severe heart, lung, kidney, brain or liver diseases; (7) malignant tumors; (8) major mental illness; (9) communication disorders; (10) other serious comorbidities and (11) unconsciousness. This present questionnaire exhibited around 80% of sensitivity and specificity, similar with that previously reported (Jones et al., 2009). To investigate potential risk factors for GERD which reflect the Tibetan lifestyle, altitude of residence, duration of residence and dietary habits were included. Detailed items of the demographic questions were listed in Table 1.

**Table 1:** Demographics and characteristics between respondents with and without GERD (*n* = 5,680).

Variables	With GERD (*n* = 614)	Without GERD (*n* = 5,066)	*P*-value
BMI (kg/m^2^)^1^	21.55 ± 5.64	21.55 ± 5.72	0.983
Age, *n* (%)^2^	40.84 ± 13.57	38.63 ± 16.09	<0.001*
Under 18 years	54 (8.8%)	907 (17.9%)	<0.001*
18–30 years	101 (16.4%)	1,260 (24.9%)	
30–40 years	233 (37.9%)	1,235 (24.4%)	
40–50 years	179 (29.2%)	894 (17.6%)	
Over 50 years	47 (7.7%)	770 (15.2%)	
Gender, *n* (%)^2^			0.210
Male	354 (57.7%)	2,786 (55.0%)	
Female	260 (42.3%)	2,280 (45.0%)	
Education level, *n* (%)^2^			<0.001*
Primary	139 (22.6%)	1,397 (27.6%)	
High school	305 (49.7%)	2,824 (55.7%)	
University	170 (27.7%)	845 (16.7%)	
Ethnic group, *n* (%)^2^			<0.001*
Tibetan	398 (64.8%)	1,691 (33.4%)	
Han	114 (18.6%)	1,965 (38.8%)	
Others	102 (16.6%)	1,410 (27.8%)	
Altitude of residence, *n* (%)^2^			<0.001*
2.5–3.0 km	59 (9.6%)	578 (11.4%)	
3.0–3.5 km	171 (27.9%)	1,725 (34.1%)	
3.5–4.0 km	268 (43.6%)	2,071 (40.9%)	
4.0–4.5 km	116 (18.9%)	692 (13.7%)	
Length of Residence, *n* (%)^2^			<0.001*
<5 years	338 (55.0%)	2,144 (42.3%)	
≥5 years	276 (45.0%)	2,922 (57.7%)	
Drinking, *n* (%)^2^			
Coffee	280 (45.6%)	2,146 (42.4%)	0.125
Buttered tea	336 (54.7%)	2,808 (55.4%)	0.740
Sweet tea	427 (69.5%)	2,527 (49.9%)	<0.001*
Alcohol, *n* (%)^2^			
Beer	312 (50.8%)	2,539 (50.1%)	0.745
White wine	289 (47.1%)	2,515 (49.6%)	0.228
Red wine	277 (45.1%)	2,129 (42.0%)	0.144
Barley wine	332 (54.1%)	2,477 (48.9%)	0.015*
Smoking, *n* (%)^2^			0.837
Yes	306 (49.8%)	2,519 (49.7%)	
No	308 (50.2%)	2,547 (50.3%)	
Dried meat, *n* (%)^2^			0.042*
Yes	335 (54.6%)	2,522 (49.8%)	
No	279 (45.4%)	2,544 (50.2%)	
Staying up late, *n* (%)^2^			0.021*
Yes	334 (54.4%)	2,506 (49.5%)	
No	280 (45.6%)	2,560 (50.5%)	
No breakfast, *n* (%)^2^			0.735
Yes	307 (50.0%)	2,499 (49.3%)	
No	307 (50.0%)	2,567 (50.7%)	
Family history of GERD, *n* (%)^2^			0.084
Yes	326 (53.1%)	2,503 (49.4%)	
No	288 (46.9%)	2,563 (50.6%)	
Medical history, *n* (%)^2^			
Pharyngitis	309 (50.3%)	2,494 (49.2%)	0.608
Asthma	294 (47.9%)	2,541 (50.2%)	0.287
Precordial pain	311 (50.7%)	2,549 (50.3%)	0.875
Constipation	291 (47.4%)	2,620 (51.7%)	0.043*
Indigestion	364 (59.3%)	2,587 (51.1%)	<0.001*
Diabetes mellitus	30 (4.9%)	287 (5.7%)	0.427
Tuberculosis	299 (48.7%)	2,515 (49.6%)	0.657
Hydatid disease	318 (51.8%)	2,569 (50.7%)	0.613
Hepatitis B	288 (49.3%)	2,385 (51.3%)	0.376
Gastric disease, *n* (%)^2^			0.322
Polypus	164 (26.7%)	1,246 (24.6%)	
Superficial gastritis	167 (27.2%)	1,284 (25.3%)	
Reflux gastritis	140 (22.8%)	1,241 (24.5%)	
Ulcer	143 (23.3%)	1,295 (25.6%)	

**Notes:**

*P*-values were based on ^1^independent two sample *t*-test and ^2^Chi-square.

Data were displayed as ^1^mean ± standard deviation or ^2^numbers (percentage).

An asterisk indicated a significant difference between the two groups (*P* < 0.05).

### Statistical analysis

For categorical variables, comparisons between subjects with and without GERD were performed using Chi-square test. Continuous variables were compared using the independent two-sample *t*-test. Descriptive statistics are reported as number and percentage for categorical variables or means with standard deviations for continuous variables. Logistic regression were performed to estimate the odds ratio (OR) and its 95% confident interval (CI) for the factors associated with GERD by constructing univariate and multivariate models. Variables with a *P*-value <0.2 in the univariate logistic regression were included in the multivariate analysis with a stepwise backward conditional method. All statistical assessments were two-sided and a *P* < 0.05 was considered statistically significant. Statistical analyses were performed using SPSS 22.0 statistics software (IBM Corp., Armonk, NY, USA).

## Results

A cross-sectional study was carried out on a sample of 10,000 individuals in the Tibet Autonomous Region in China using a previously validated questionnaire (GROUP, 2004; Jones et al., 2009). From the 10,000 distributed questionnaires, 6,102 were retrieved. Among them, 422 were excluded due to incomplete response, and there were no drop-outs. Therefore, 5,680 completed questionnaires were analyzed, 3,140 (55.3%) were men. Mean age of respondents was 38.9 ± 15.9 years while the mean body mass index (BMI) was 21.5 ± 5.7 kg/m^2^. Demographic characteristics of responders are shown in Table 1. A total of 614 patients (10.8%) were diagnosed as GERD by using the questionnaire. There were significant differences in terms of age, education level, ethnic group, altitude of residence, length of residence, drinking sweet tea and barley wine, eating dried meat, staying up late, and having medical history of constipation and indigestion between respondents with and without GERD (all *P* < 0.05).

Table 2 presented the results of univariate logistic regression analysis to determine the risk factors potentially associated with GERD. The univariate logistic regression model indicated the age, education level, ethnic group, altitude of residence, length of residence, sweet tea, barley wine, dried meat, and staying up late were associated with GERD (*P* < 0.05). Moreover, variables having a *P*-value < 0.2 in the univariate analysis were selected and evaluated by multivariate logistic regression models with a stepwise backward conditional process.

**Table 2:** Univariate logistic regression analysis on the factors associated with GERD.

Variables	OR [95% CI]	*P*-value
Age		
18–30 years vs. Under 18 years	1.346 [0.957–1.894]	0.088
30–40 years vs. Under 18 years	3.169 [2.328–4.313]	<0.001*
40–50 years vs. Under 18 years	3.363 [2.447–4.622]	<0.001*
Over 50 years vs. Under 18 years	1.025 [0.685–1.533]	0.903
BMI	1.000 [0.986–1.015]	0.983
Gender		
Male vs. Female	1.114 [0.941–1.320]	0.211
Education level		
High school vs. Primary	1.114 [0.941–1.320]	0.445
University vs. Primary	2.022 [1.591–2.570]	<0.001*
Ethnic group		
Han vs. Tibetan	0.246 [0.198–0.307]	<0.001*
Others vs. Tibetan	0.307 [0.245–0.386]	<0.001*
Altitude of residence, *n* (%)		
3.0–3.5 km vs. 2.5–3.0 km	0.971 [0.712–1.325]	0.853
3.5–4.0 km vs. 2.5–3.0 km	1.268 [0.942–1.705]	0.117
4.0–4.5 km vs. 2.5–3.0 km	1.642 [1.178–2.290]	0.003*
Length of residence		
<5 years vs. ≥5 years	1.669 [1.410–1.975]	<0.001*
Coffee		
Yes vs. No	1.141[0.964–1.350]	0.125
Buttered tea		
Yes vs. No	0.972 [0.821–1.150]	0.740
Sweet tea		
Yes vs. No	2.294 [1.915–2.748]	<0.001*
Beer		
Yes vs. No	1.028 [0.870–1.216]	0.745
White wine		
Yes vs. No	0.902 [0.763–1.067]	0.228
Red wine		
Yes vs. No	1.134 [0.958–1.342]	0.144
Barley wine		
Yes vs. No	1.231 [1.040–1.456]	0.016*
Smoking		
Yes vs. No	0.983 [0.831–1.162]	0.837
Dried meat		
Yes vs. No	1.190 [1.006–1.408]	0.042*
Stay up late		
Yes vs. No	1.219 [1.030–1.442]	0.021*
No breakfast		
Yes vs. No	1.027 [0.869–1.215]	0.753
Family history of GERD		
Yes vs. No	1.159 [0.980–1.371]	0.085
Pharyngitis		
Yes vs. No	1.045 [0.884–1.235]	0.608
Asthma		
Yes vs. No	0.913 [0.772–1.080]	0.287
Diabetes mellitus		
Yes vs. No	0.855 [0.582–1.258]	0.427
Tuberculosis		
Yes vs. No	0.963 [0.814–1.138]	0.657
Hydatid disease		
Yes vs. No	1.044 [0.883–1.235]	0.613
Hepatitis B		
Yes vs. No	0.925 [0.779–1.099]	0.376
Gastric disease		
Ulcer vs. Polypus	0.988 [0.786–1.243]	0.919
Superficial gastritis vs. Polypus	0.857 [0.675–1.088]	0.206
Reflux gastritis vs. Polypus	0.839 [0.662–1.064]	0.147

**Note:**

An asterisk indicated the significant risk factor (*P* < 0.05).

The results from the multivariate analysis showed that age (30–40 years vs. under 18 years, OR: 3.025 (95% CI [2.147–4.261]), *P* < 0.001; 40–50 years vs. under 18 years, OR: 4.484 (95% CI [3.145–6.393]), *P* < 0.001), education level (high school vs. primary, OR: 0.698 (95% CI [0.550–0.885]), *P* = 0.003; university vs. primary, OR: 2.804 (95% CI [2.090–3.761]), *P* < 0.001), ethnic group (Han vs. Tibetan, OR: 0.230 (95% CI [0.183–0.289]), *P* < 0.001; others vs. Tibetan, OR: 0.304 (95% CI [0.239–0.386]), *P* < 0.001), altitude of residence (4.0–4.5 km vs. 2.5–3.0 km, OR: 2.469 (95% CI [1.714–3.556], *P* < 0.001), length of residence (<5 years vs. ≥5 years, OR: 2.218 (95% C.I [1.836–2.679]), *P* < 0.001), sweet tea (yes vs. no, OR: 2.158 (95% CI [1.782–2.613]), *P* < 0.001), barley wine (yes vs. no, OR: 1.271 (95% CI [1.060–1.523]), *P* = 0.009), dried meat (yes vs. no, OR: 1.278 (95% CI [1.067–1.532]), *P* = 0.008) and staying up late (yes vs. no, OR: 1.229 (95% CI [1.026–1.472]), *P* = 0.025) were independently associated with GERD (Table 3).

**Table 3:** Multivariate logistic regression analysis on the risk factors for GERD.

Variables	OR [95% CI]	*P*-value
Age		
18–30 years vs. Under 18 years	0.815 [0.553–1.202]	0.302
30–40 years vs. Under 18 years	3.025 [2.147–4.261]	<0.001*
40–50 years vs. Under 18 years	4.484 [3.145–6.393]	<0.001*
Over 50 years vs. Under 18 years	1.128 [0.731–1.739]	0.557
Education level		
High school vs. Primary	0.698 [0.550–0.885]	0.003*
University vs. Primary	2.804 [2.090–3.761]	<0.001*
Ethnic group		
Han vs. Tibetan	0.230 [0.183–0.289]	<0.001*
Others vs. Tibetan	0.304 [0.239–0.386]	<0.001*
Altitude of residence, *n* (%)		
3.0–3.5 km vs. 2.5–3.0 km	1.174 [0.842–1.635]	0.344
3.5–4.0 km vs. 2.5–3.0 km	1.275 [0.926–1.756]	0.136
4.0–4.5 km vs. 2.5–3.0 km	2.469 [1.714–3.556]	<0.001*
Length of residence		
<5 years vs. ≥5 years	2.218 [1.836–2.679]	<0.001*
Sweet tea		
Yes vs. No	2.158 [1.782–2.613]	<0.001*
Barley wine		
Yes vs. No	1.271 [1.060–1.523]	0.009*
Dried meat		
Yes vs. No	1.278 [1.067–1.532]	0.008*
Staying up late		
Yes vs. No	1.229 [1.026–1.472]	0.025*
Family history of GERD		
Yes vs. No	1.173 [0.979–1.406]	0.083

**Note:**

An asterisk indicated the significant risk factor (*P* < 0.05).

## Discussion

Our present work was the first large-scale epidemiologic study of GERD in the Tibet Autonomous Region, China. The overall prevalence of GERD in this population was 10.8% (614/5,680), which was higher than that (~5%) reported in previous studies conducted in China (Chen et al., 2005; He et al., 2010; Hu et al., 2002; Pan et al., 2008; Wong et al., 2003). Further analysis showed that Tibetans (398/2,089, 19.1%) are significantly at higher risk for GERD than Chinese Han people (114/2,079, 5.5%; Han vs. Tibetan, OR: 0.231) and other ethnic populations (102/1,512, 6.7%; others vs. Tibetan, OR: 0.306). Several risk factors associated with the unique dietary habits in the Tibet Autonomous Region, including Tibetan sweet tea, barley wine and dried meat, were identified. Although the role of dietary habit in GERD remains controversial (Festi et al., 2009; Kaltenbach, Crockett & Gerson, 2006; Sethi & Richter, 2017), individuals who have Tibetan foods were at increased risk for GERD in this population. In addition, staying up late was identified as a risk factor for GERD; on the other hand, smoking, no breakfast or consumption of a variety of beverages (except Tibetan ones) were not significantly associated with GERD in this study, suggesting that lifestyle and dietary habits are related to GERD pathogenesis (Meining & Classen, 2000; Ness-Jensen et al., 2016; Yamamichi et al., 2012). It is interesting that Tibetan barley wine but other alcoholic beverages in the questionnaire are significantly associated with GERD. Considering that there is no special brewing procedure or higher alcohol by volume of Tibetan barley wine, it is possible that certain unidentified molecules and/or its fermentation product(s) in Tibetan barley, the principal cereal cultivated on the Tibetan Plateau, induce or exacerbate GERD.

A previous study conducted in India reported that the prevalence of GERD in a high altitude area (≥3,000 m) is 18.7% (Kumar et al., 2011), which was not significantly different from that in either northern (16.2%) (Sharma et al., 2011) or southern (22.2%) (Wang et al., 2016) India. Although higher altitude of residence did not increase the risk of GERD in Indian population, it was identified as a risk factor of GERD in this study. It is worth to note that the prevalence of GERD in India (16.2–22.2%) is much higher than that in China (<10%); therefore, there might be hidden factor(s) that dominates the altitude factor. Moreover, it is noted that people with shorter duration of residence is at higher risk for GERD. Both observations implied that a stringent geographic condition is a risk factor for GERD which may be overcome by adaptation. Further studies may focus on the association between GERD with the changed atmospheric pressure and oxygen concentration and/or large differences in day and night temperature.

Due to the unique geography of the Tibetan Plateau, the Tibet Autonomous Region is a rural area compared with first-tier cities in China. However, rapid urbanization occurs in this area within decades. Higher education level and staying up late, which are considered as indicators of urbanization, were associated with GERD. It was also noted that the employed population is at higher risk for GERD. Compared with people ≤18 (54/961, 5.6%) and ≥50 (47/817, 5.8%) years of age, the prevalence of GERD was higher in people with 30–50 (30–40 years: 233/1,468, 15.9%, OR = 3.047; 40–50 years: 179/1,073, 16.7%, OR = 4.462) years of age, and an increased trend was observed in people with 18–30 (101/1,361, 7.4%) years of age, reflecting that pressure from working and/or family may increase the risk for GERD. Family history of GERD was a risk factor identified in several large-scale studies (El-Serag et al., 2004; He et al., 2010; Locke et al., 1999; Mohammed et al., 2003); however, it was not identified as a risk factor in this present study. Also, respiratory, cardiovascular, gastrointestinal, infectious diseases or diabetes mellitus, was not associated with GERD among the medical history questions in our series. It is interesting that high BMI or diabetes was not a risk factor for GERD in the Tibet Autonomous Region. One possibility is that Tibetans has adapted to the high-altitude environment and show several distinct genotypes and phenotypes compared to other populations (Yang et al., 2017). Also, Tibetans adapt to the stringent geological condition by developing the unique Tibetan lifestyle. Although there are no large-scale studies focusing on the BMI among Tibetans to date, a recent study has reported that the prevalence of diabetes among Tibetans in China is 2.9% (NCD Risk Factor Collaboration (NCD-RisC), 2016), which is quiet lower than that (11.1–12.3%) in middle-income countries (Dagenais et al., 2016). The ethnic difference and unique lifestyle may be two of the reasons that high BMI and diabetes were not identified as risk factors for GERD in this study. Also, further studies focused on the distinct features of higher BMI/diabetes patients in Tibet vs. in other areas would be helpful for explaining this observation.

The prevalence of GERD was higher in the Tibet Autonomous Region than in other regions in China. Based on the risk factors identified in this study, reducing Tibetan dietary, including sweet tea, barley wine and dried meat, and avoiding staying up late would be helpful for GERD management and/or prevention in the Tibet Autonomous Region.

There were several limitations in this study. This cross-sectional study was difficult to further analyze temporal relationships between risk factors and GERD. Due to the limited medical resources in the Tibet Autonomous Region, the questionnaires were mainly recovered from the responders who were invited to answer in the hospitals in prefecture-level cities, which may only reflect the epidemiology of GERD in the urban but rural areas of the Tibet Autonomous Region. Other typical or atypical symptoms which were not included in the questionnaire may lead to underestimation of the GERD prevalence in this region.

## Conclusion

The prevalence of GERD in people living in the Tibet Autonomous Region was higher than that reported from other large-scale China studies. The unique dietary habits and the extreme geographic conditions in the Tibet Autonomous Region increased the risk of GERD. Tibetan ancestry, urbanization and gastrointestinal symptoms were risk factors for GERD.

## Supplemental Information

10.7717/peerj.6491/supp-1 Supplemental Information 1 Raw data.

## Additional Information and Declarations

### Data Availability

The following information was supplied regarding data availability: The raw measurements are available in the Supplemental File.
